# Is the message getting through? Awareness and use of the 11+ injury prevention programme in amateur level football clubs

**DOI:** 10.1371/journal.pone.0195998

**Published:** 2018-04-19

**Authors:** Jan Wilke, Daniel Niederer, Lutz Vogt, Winfried Banzer

**Affiliations:** Goethe University Frankfurt am Main, Department of Sports Medicine, Frankfurt am Main, Germany; Universite de Nantes, FRANCE

## Abstract

A large body of evidence suggests that the 11+ warm-up programme is effective in preventing football-related musculoskeletal injuries. However, despite considerable efforts to promote and disseminate the programme, it is unclear as to whether team head coaches are familiar with the 11+ and how they rate its feasibility. The present study aimed to gather information on awareness and usage among German amateur level football coaches. A questionnaire was administered to 7893 individuals who were in charge of youth and adult non-professional teams. Descriptive and inferential statistics were used to analyse the obtained data. A total of 1223 coaches (16%) returned the questionnaire. There was no risk of a non-response bias (p>.05). At the time of the survey, nearly half of the participants (42.6%) knew the 11+. Among the coaches who were familiar with the programme, three of four reported applying it regularly (at least once per week). Holding a license (φ = .28, p < .0001), high competitive level (Cramer-V = .13, p = .007), and coaching a youth team (φ = .1, p = .001) were associated with usage of 11+. Feasibility and suitability of the 11+ were rated similarly by aware and unaware coaches. Although a substantial share of German amateur level coaches is familiar with the 11+, more than half of the surveyed participants did not know the programme. As the non-usage does not appear to stem from a lack of rated feasibility and suitability, existing communication strategies might need to be revised.

## Introduction

Warming up is a well-established method to prepare for the physical demands of sports and exercise. Its main benefits encompass a reduction of injury risk [1] and an increase in motor performance [2]. As the physiological requirements and types of injury vary substantially depending on the respective sports [3–6], specific warm-up programmes, tailored to account for the individual load compositions and biomechanics, have been proposed. The ‘11+’ was developed for football (soccer) players [7]. It consists of 15 progressive exercises and is divided into three sections (part 1: slow running and active stretching, part 2: core and leg stability, balance, plyometrics, and agility, part 3: fast running and cutting movements). The 20-minute-intervention is intended to be performed two or more times per week prior to practice and matches.

According to a series of systematic reviews and meta-analyses, the application of 11+ decreases the overall number of injuries by 35 to 39% [8–10]. Several studies, moreover, report that high compliance with the 11+ further reduces the odds of sustaining an injury [6,11,12]. In view of these findings, considerable efforts have been made to facilitate the transfer into practice. The programme is available in four languages (English, Spanish, German, French) and the International Federation of Association Football (FIFA) advertised its use by means of scientific lectures, workshops, video materials, and printed manuals in more than 70 countries [13,14]. In Germany, which has been described as an implementation model, the FIFA has developed an extensive dissemination plan, collaborating with both, the national football association and one of the national insurance organisations [15]. However, it is unclear as to how effective the several strategies, aiming to promote the 11+, have been so far.

Owoeye et al. [16] elucidated the knowledge of 11+ in Nigeria. Their survey was administered to athletes only, which is of relevance because the team coaches and health professionals represent the decisive stakeholders for implementation [15]. Al Attar et al. [17] surveyed Australian and Saudi-Arabian coaches, but awareness was not assessed. In contrast, O’Brien et al. [18] explicitly assessed this variable, reporting that 61% of the study participants knew the 11+. Yet, their sample was a) a mix of coaches and health professionals, and b), with 18 participants, very small. Against this background, the present study aimed to provide a population-based, cross-sectional overview of awareness and usage of the 11+ in German amateur level football coaches.

## Materials & methods

### Study design and ethical considerations

An electronic web-based cross-sectional survey was performed between September 2015 and June 2016. The study followed the recommendations for *Good Practice in the Conduct and Reporting of Survey Research* [19]. Ethical approval was obtained from the local committee (Ethics Committee of the Faculty of Psychology and Sports Sciences at Goethe University Frankfurt, Germany) and all participants gave digital informed consent. After being provided with information on aims, content, anonymous data collection and data processing on the first page of the questionnaire, they were explicitly advised that by clicking the “continue” button, they would agree to participate in the study, accepting the aforementioned terms.

### Sample

Germany has a hierarchical league system, which is characterised by the possibility of being promoted (division up) or relegated (division down) for the next season in dependency of performance. Besides the first four professional divisions, the national football association (Deutscher Fussball-Bund, DFB) has established an extensive, highly organised amateur football scene, including up to 8 divisions (5^th^ to 12^th^) in each of its 21 regional sub-associations. Within the FIFA, the DFB represents the largest member organisation worldwide [15].

The target population of our study comprised participants coaching male youth (12 to 18 years) or adult amateur level teams. Hence, for the present study, the coaches of all clubs, except for those in the first four professional or semi-professional divisions, were eligible. To recruit participants, the 21 official, regional football associations of the country were invited to collaborate by means of personal communication and e-mail. The participating associations were sent an electronic letter containing both a comprehensive description of the study’s aims and contents as well as the link to the online questionnaire. The associations, then, distributed the questionnaire to amateur football coaches within their area of responsibility using their respective mailing list. In accordance with relevant guidelines, a reminder was sent to all participants after seven days [20].

### Questionnaire

The used questionnaire was created based on the approach of a similar previous study [16]. After a thorough literature review, a group consensus process was initiated. To agree on scope and content, all authors met and gathered ideas. Following the meeting, one investigator created a basic version of the questionnaire which was edited according to a discussion about the other authors’ feedback. Subsequently, for face validation, the questionnaire was sent to movement scientists and physicians, who were not familiar with the project. After once again adapting the items, using their feedback, the questionnaire was administered a) to two football coaches without any scientific background and b) to two officials of a cooperating local football association.

The two coaches were current license holders, supervising teams playing on an amateur level, analogous to the target sample. The officials were responsible for the license education on the amateur level and, therefore, also had proficient knowledge regarding the investigated sample. All four individuals involved in the pilot testing reported good comprehensibility and preciseness of the questionnaire, making only minor suggestions for improvement. The final instrument is displayed in *Table 1.* Its first part assessed general aspects and responsibilities (e.g., license possession, employment of a conditioning coach, training frequency, or suspected purposes of warm-up), while the second part addressed specific questions with regard to awareness and usage of 11+.

**Table 1:** Contents of the survey administered to the football coaches.

Question	Answer format
Is your team a youth or an adult team?	single choice
In which league does your team play?	single choice
Do you have a coaching license?	yes/no
Do you have a strength and conditioning coach?	yes/no
How many training sessions does your team have per week?	free entry
What is the usual duration of one training session?	free entry
What is the duration of your warm-up prior to practice?	free entry
What is the duration of your warm-up prior to matches?	free entry
How important are the following aspects on a scale from 0 (not important) to 10 (very important) with regard to warming up? • Reduction of injury risk • Increase of motor performance • Mental preparation	Likert scales (10 items)
Which of these standardised warm-up programmes do you know? • 11+ • PEP (Prevent Injury and Enhance Performance) • KIPP (Knee injury prevention program) • Others • None	multiple choice + free entry
*If 11+ was selected:*	
Do you use the 11+ in your team?	yes/no
How often does your team perform the 11+ per week prior to training?	multiple choice
Does your team perform the 11+ prior to matches?	yes/no
How suitable do you rate the 11+ for your football players on a scale from 0 (not suitable) to 10 (very suitable)?	Likert scale (10 items)
How feasible do you rate the 11+ for your training on a scale from 0 (not feasible) to 10 (very feasible)?	Likert scale (10 items)
If 11+ was not selected (programme unknown)*:	
Can you imagine using the 11+ as your future warm-up? • Yes • No • I already use similar exercises	single choice
How suitable do you rate the 11+ for your football players on a scale from 0 (not suitable) to 10 (very suitable)?	Likert scale (10 items)
How feasible do you rate the 11+ for your training on a scale from 0 (not feasible) to 10 (very feasible)?	Likert scale (10 items)

*: If the participants did not know the 11+, a flyer describing and depicting the exercises was displayed.

### Data processing and statistics

To assess the risk of a non-response bias, a wave analysis was performed according to Lewis et al. [21]. The wave analysis is based on the assumption that quick responders are more motivated to participate, while participants who answer late can be considered similar to non-responders. To test for systematic differences between these groups, a chi squared test was calculated comparing the first 10% of the data obtained (early responders) to the last 10% (late responders). Tested variables were (1) awareness of 11+, (2) application of 11+, and (3) readiness to implement 11+ in the future among users unaware of the programme.

Following risk of bias testing, descriptive and inferential statistics were used to analyse the obtained data, as appropriate. Interval scaled and dichotomous data (e.g., awareness, use, readiness for implementation in non-users) were reported as absolute (n) and relative (%) values, respectively. For nominal and ordinal scaled results (suitability, feasibility, purposes of warm-up), medians plus first and third quartiles (Q1/Q3) were calculated. To detect systematic associations of the assessed variables (e.g., between license possession/ level of play/age class/purpose of warm-up and use/ awareness/ readiness), point biserial correlations (binary and (quasi)-interval variables) and contingency tables (phi coefficients for dichotomous nominal data respectively Cramer’s V for polytomous nominal data) were calculated.

Data analysis software SPSS 22 (SPSS Inc., Chicago, Illinois USA) was used for all analyses and significant associations among study variables were inferred at α = .05.

## Results

**Fig 1:**
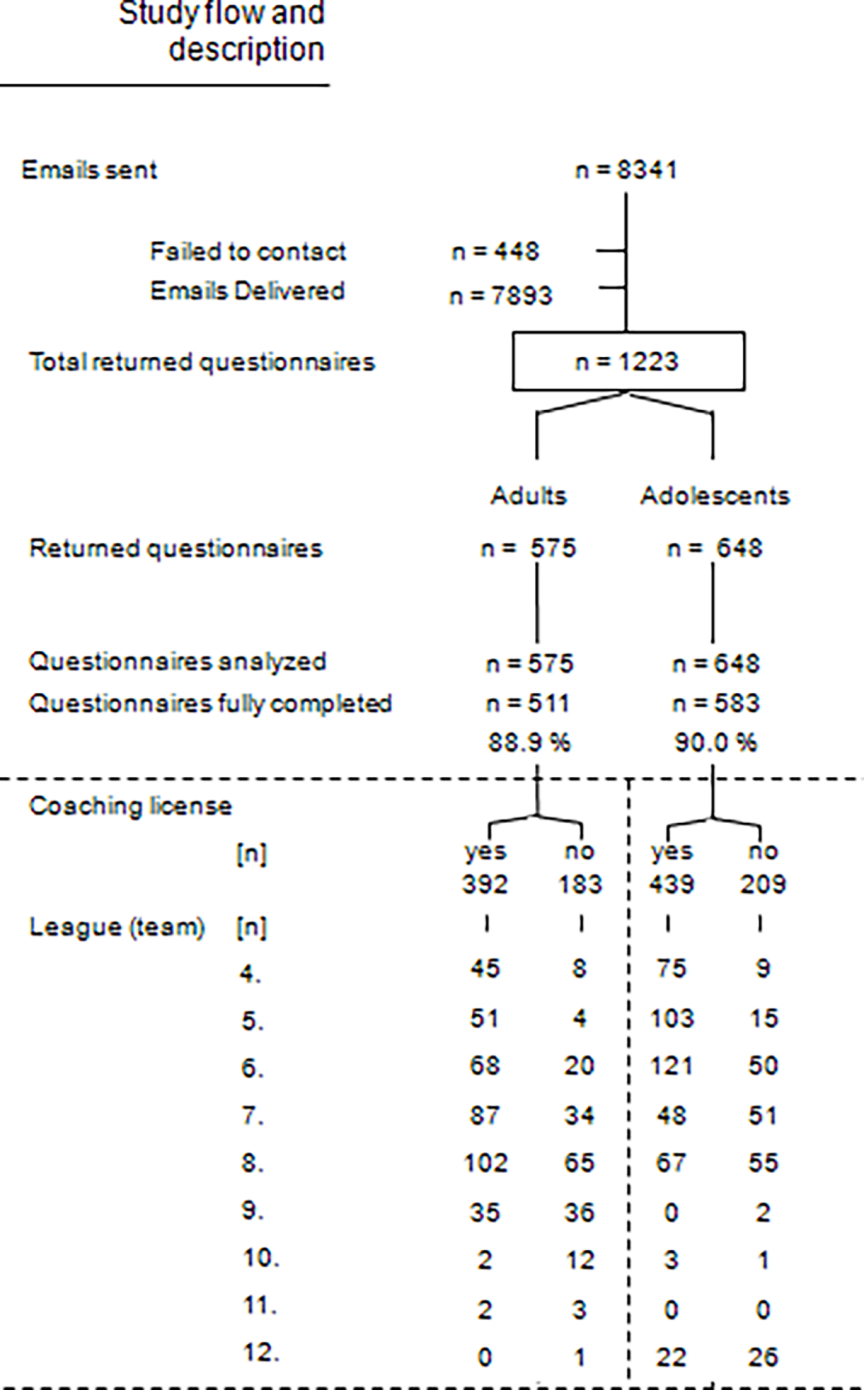
Depiction of the study flow.

Out of 7893 emailed coaches, 1223 (16%) completed the survey. According to the wave analysis, there was no significant non-response bias in terms of the three variables tested (p>.05). The study flow is depicted in Fig 1.

### Responsibilities and general warm-up-routines

Slightly more than half of the respondents (53%, 648/1223) were in charge of youth teams while 47% coached an adult team. About two thirds (67.9%, n = 830) of the participants reported holding an official coaching license and 9.6% (n = 117) of the teams have a strength and conditioning coach. Practice frequency was 2.4±0.6 sessions per week with a total duration of 92.7±18.4 minutes per session. The estimated warm-up duration was 19.4±6 minutes. Prior to matches, warm-up time was 27.1±8 minutes. According to the coaches’ rating, the most important purpose of warm-up was injury prevention (median 10, Q1: 9, Q3: 10). Both mental preparation (Q1:7, Q3: 8) and increasing performance (Q1:6, Q3: 9) were rated 8 on the Likert scale.

### Awareness and usage of the 11+ programme

With 42.6% (521/1223 coaches), nearly the half of the surveyed participants knew the 11+ at the time of the survey, and 32.1% (n = 393) reported using it. Among the coaches who were aware of the programme, 75.4% (393/521) indicated application.

Most of the 11+ users (95.1%, 374/393) perform the 11+ exercises regularly (at least once a week) during practice, but only 40.8% (n = 160) apply it twice a week as recommended by the developers. Also, only slightly more than half (56.6%, n = 222) of the 11+ users have implemented the programme in pre-game warm-up. Using the 11+ was not associated with the number of training sessions per week (p = .11) or the duration of the warm-up (p = .94) prior to practice and matches.

Both suitability and feasibility were rated similarly high by both, aware and unaware coaches (*Table 2*). 43.7% of the latter (307/702) consider implementing it in the future, while 6.8% (n = 48) were not interested in using it. The remaining 50.1% (n = 352) reported already applying similar exercises during warm-up.

**Table 2:** Coaches’ ratings of suitability and feasibility.

	Ratings per category [pts]										GroupMedian
	*1*	*2*	*3*	*4*	*5*	*6*	*7*	*8*	*9*	*10*	
Aware, using 11+	-	1 (0.3)	2 (0.5)	2 (0.5)	10 (2.6)	22 (5.7)	65 (16.9)	141 (36.6)	72 (18.7)	65 (16.9)	8 (7/9)
Aware, not using 11+	1 (0.7)	2 (1.5)	4 (2.9)	7 (5.1)	14 (10.2)	22 (16.1)	27 (19.7)	40 (29.2)	13 (9.5)	6 (4.4)	7 (6/8)
Not aware of 11+	6 (0.9)	5 (0.7)	14 (2.1)	17 (2.5)	44 (6.5)	49 (7.2)	135 (19.8)	200 (29.3)	96 (14.1)	82 (12.0)	8 (7/9)
	Ratings per category [pts]										GroupMedian
	*1*	*2*	*3*	*4*	*5*	*6*	*7*	*8*	*9*	*10*	
Aware, using 11+	-	1 (0.3)	4 (1.0)	6 (1.6)	16 (4.2)	26 (6.8)	57 (14.8)	117 (30.4)	79 (20.5)	73 (19)	8 (7/9)
Aware, not using 11+	2 (1.5)	4 (2.9)	2 (1.5)	5 (3.6)	8 (5.8)	20 (14.6)	19 (13.9)	44 (32.1)	14 (10.2)	16 (11.7)	8 (6/8)
Not aware of 11+	6 (0.9)	6 (0.9)	15 (2.2)	14 (2.1)	36 (5.3)	57 (8.4)	102 (15.0)	166 (24.3)	112 (16.4)	126 (18.5)	8 (7/9)

Data assessed with a likert scale (1 to 10 points, small values: poor suitability/feasibility). Ratings per category are absolute frequencies plus percent values (brackets). For the group medians, the 1^st^ and 3^rd^ quartile are shown in brackets; Aware users: n = 380, aware non-users: n = 136, unaware coaches: n = 648.

Knowledge of the programme was not associated with the perceived importance of preventing injury (p = .5), increasing performance (p = .71) or better mental preparation (p = .11) through warm-up procedures (*Table 3*). However, among the coaches who were familiar with the programme, there was a correlation of 11+ usage and the rated importance of injury prevention by means of warming up (r = .13, p = .003). Factors being positively correlated with knowledge of the 11+ programme encompassed holding a coaching license (φ = .28, p < .0001), high competitive level (Cramer-V = .13, p = .007), and coaching a youth team (φ = .1, p = .001). In addition, there was a tendency for higher awareness of the 11+ if the coached team employed a strength and conditioning coach (φ = .05, p = .07).

**Table 3:** Coaches’ ratings of the importance of different warm-up purposes.

Injury prevention											
	Ratings per category										Group median
	*1*	*2*	*3*	*4*	*5*	*6*	*7*	*8*	*9*	*10*	
Aware , using 11+	2 (0.5)	3 (0.8)	-	1 (0.3)	3 (0.8)	10 (2.6)	11 (2.9)	51 (13.2)	51 (13.2)	253 (65.7)	10 (9/10)
Aware, not using 11+	3 (2.2)	1 (0.7)	1 (0.7)	2 (1.5)	3 (2.2)	4 (2.9)	9 (6.6)	22 (16.1)	11 (8.0)	81 (59.1)	10 (8/10)
Not aware of 11+	5 (0.7)	5 (0.7)	6 (0.9)	5 (0.7)	12 (1.8)	16 (2.3)	28 (4.1)	83 (12.2)	109 (16.0)	413 (60.6)	10 (9/10)
Performance enhancement											
	Ratings per category										Group median
	*1*	*2*	*3*	*4*	*5*	*6*	*7*	*8*	*9*	*10*	
Aware, using 11+	4 (1.0)	10 (2.6)	14 (3.6)	21 (5.5)	37 (9.6)	34 (8.8)	56 (14.5)	68 (17.7)	58 (15.1)	83 (21.6)	8 (7/8)
Aware, not using 11+	6 (4.4)	4 (2.9)	11 (8)	13 (9.5)	7 (5.1)	9 (6.6)	18 (13.1)	22 (16.1)	19 (13.9)	28 (20.4)	8 (4.5/8)
Not aware of 11+	4 (1.0)	10 (2.6)	14 (3.6)	21 (5.5)	37 (9.6)	34 (8.8)	56 (14.5)	68 (17.7)	58 (15.1)	83 (21.6)	8 (7/8)
Mental preparation											
	Ratings per category										Group median
	*1*	*2*	*3*	*4*	*5*	*6*	*7*	*8*	*9*	*10*	
Aware, using 11+	2 (0.5)	2 (0.5)	9 (2.3)	7 (1.8)	24 (6.2)	18 (4.7)	55 (14.3)	94 (24.4)	64 (16.6)	110 (28.6)	8 (7/8)
Aware, not using 11+	-	-	1 (0.7)	4 (2.9)	4 (2.9)	13 (9.5)	13 (9.5)	40 (29.2)	20 (14.6)	42 (30.7)	8 (7/8)
Not aware of 11+	2 (0.5)	2 (0.5)	9 (2.3)	7 (1.8)	24 (6.2)	18 (4.7)	55 (14.3)	94 (24.4)	64 (16.6)	110 (28.6)	8 (6/9)

Data assessed with a likert scale (1 to 10 points, small values: low importance). Ratings per category are absolute frequencies plus percent values (brackets). For the group medians, the 1^st^ and 3^rd^ quartile in shown in brackets. Aware users: n = 385, aware non-users: n = 137, unaware coaches: n = 682.

Among the coaches who were unaware of the programme, non-possession of a license was associated with an increased readiness to use the 11+ in the future (Cramer-V: .14, p = .003). In contrast, level of play (p = .16), age class (youth or adult teams, p = .93) and training volume (unit duration: p = .71, units per week: p = .15) did not influence readiness.

## Discussion

Although a large body of evidence supports the effectiveness of the 11+ and great efforts have been made to disseminate the intervention [8,13], the present study is the first to systematically investigate its awareness and use in a large population-based sample of amateur level football coaches. Considering that they represent a major stakeholder in decision-making with regard to the implementation of injury prevention programmes, gathering information on their attitudes and beliefs is crucial.

### Awareness of the 11+

Four out of ten coaches knew the 11+ at the time of the survey, which is in slight contrast to the results of previous studies. Investigating the success of implementation of an earlier version of the programme (‘The 11’) in Switzerland, Junge et al. [22] found an awareness level of 80%. However, the study included an initial education of all participating coaches, and thus, the obtained may not be representative of the total population. With a share of 61%, also O’Brien et al. [18] found a higher degree of knowledge than our survey. This discrepancy could be explained by a variety of factors, e.g., the mixed study population (coaches, physiotherapists and fitness trainers) and the small sample size recruited by O’Brien and colleagues. In their study, Owoeye et al. [16] revealed that only 20.7% of the Nigerian youth players knew the programme. As coaches rather than athletes represent the programme deliverer, the lower degree of awareness seems plausible.

Although a considerable share of the football coaches included in our study knew the 11+, the effectiveness of existing strategies, aiming to promote the programme, might be improved. One possibility to achieve greater knowledge consists in coach education. In accordance to the standards set by the Union of European Football Association (UEFA), Germany offers a progressive license system with four main levels (C level, B level, A level, and Pro level, [23]). An analysis of the educational systems’ contents revealed that some of the issued licenses (e.g., A level and B level) include topics specifically dedicated to fitness training and prevention. Possibly, due to this, ownership of a license was associated with higher awareness. However, a more expansive dissemination of knowledge on injury prevention programmes could represent a promising approach to further tackle the lack of awareness. This assumption is substantiated by two facts. In the present sample, non-users without a license displayed a higher readiness for implementation compared to license holders. Moreover, according to another study, coaches classify inadequate warm-up as a risk factor for injury even less frequently than do players [24].

In addition to license possession, being a coach of a higher league team was positively correlated with awareness of 11+. Although this might be a translucent correlation (coaches at lower competitive level teams hold a coaching license less frequently), the development of strategies targeting the group of coaches in lower leagues seems advisable. While optimizing license education should raise awareness in higher level teams, other methods such as workshops or brochures might be more effective in teams at lower competitive levels. With regard to the age category of the teams, 11+ is less known in coaches of adult teams when compared to youth teams, although the number of license holders was similar in both subgroups. A possible reason for this might consist of different educational contents within the licensing system.

### Use of the 11+

Almost three quarters of the aware coaches reported regular usage. This finding is very well in line with data of Al Attar et al. [17] who demonstrated almost identical values in Australian coaches (73%). Nonetheless, usage of 11+ appears to be highly region-dependent. In their study, Al Attar and colleagues also reported that, in Saudi Arabia, only 40% of the surveyed participants used the programme. Owoeye et al. [16] found an even lower share of 28% use in a Nigerian sample, but as they investigated players, their results, besides geographical location, might stem from differential perspectives of athletes and coaches.

The high usage rate in aware coaches of our study indicates that the 11+ appears to meet the needs of the surveyed participants. However, a considerable number of the coaches who knew the programme do not apply it, and more than the half of the coaches who apply it do not use it at least twice a week according to the recommendations. Similar incomplete implementation, not adopting an intervention as designed, has also been observed for other injury prevention programmes [25]. For non-usage, several factors, such as non-availability of equipment, limited space, lack of time, and lack of rated programme quality, have been discussed [26,27]. Based on the present study, assumptions can be made on two of them: available time and programme quality. Neither total practice duration nor the amount of time dedicated to warm-up prior to practice or matches were associated with programme usage. It can consequently be inferred that a lack of time does not represent a decisive factor. This is in contrast to the work of McKay et al. [26] who found that lack of time represents a main barrier for the implementation in previously unaware coaches. With regard to the suitability, it seems unlikely that non-usage stems from a lack of quality. Firstly, the coaches who did not previously know the programme rated feasibility and content-related suitability equally high as current users. It has to be underlined that coaches who already performed the 11+ judged it based on own experience derived from practical application, whereas unaware coaches did not have this possibility. Notwithstanding, the ratings represent an interesting indication for the unaware coaches’ first impression. Secondly, if not already using similar exercises, most non-users reported the readiness to incorporate 11+ in the future. It can, thus, cautiously be concluded that the contents appear to convince most of the previously unaware football coaches.

### Future directions

Several aspects connected to the findings of the present study call for further research. Based on the results, in order to increase the adherence to the 11+ or other standardised, sport-specific warm-up programmes, a distinction should be made between the goals of raising awareness in coaches unaware and facilitating usage in coaches aware. With regard to the former, it seems paramount to evaluate the effectiveness of existing communication strategies aimed at promoting injury prevention and warm-up programmes. This, for instance, could be achieved by finding out how the coaches heard from the intervention (e.g., brochure, license education, conference lectures) and which channels of information they generally rely on in order to keep on track with new methods and contents.

For the already aware coaches, a potential issue relates to the comprehensibility and clarity of the currently used communication strategies. Half of the non-users surveyed in this study reported already applying similar exercises. As most coaches have limited expertise with regard to exercise science and sports medicine, it might be that they a) in fact apply similarly effective contents, or b) do not recognise an existing difference to evidence-based programmes. In the latter case, the peculiarities and advantages of interventions like the 11+ would have to be explained in more detail or more clearly, respectively. Connecting to this, another barrier might be the self-perceived inability of the coaches to implement the programme. In a study that examined the implementation of a method teaching proper landing technique, nearly half of the interviewed coaches did not feel adequately prepared for the task [28]. Also, a lack of knowledge about adequate implementation was reported to be a major obstacle to adopting a prevention programme for anterior cruciate ligament injury [26]. If the same would apply to 11+, the didactics and contents of current education strategies should be adjusted in dependency of the coaches’ educational background.

Besides examining different communication strategies and didactics of education, it might be of importance to identify the most effective methods of programme delivery. Steffen et al. [6] demonstrated in a cluster-randomised trial that, in terms of team adherence, the involvement of a physiotherapist does not add an extra value over delivery by the coaches themselves. More research delineating the stakeholder’s relative importance (e.g. the role of the parents in youth teams) within the implementation process is warranted.

### Limitations

Some shortcomings need to be discussed. The implementation of an injury prevention programme is a complex process and the variable ‘usage’, which was assessed in our study, does not represent its only facet. Besides coaches, which were invited to participate in this survey, sports-related organisations, club officials, medical professionals and the players are other relevant actuators potentially affecting the success of implementation. Another aspect relates to the construction of the questionnaire. Prior to data collection, we performed a pilot-testing in a small sample of the target population, but there was no extensive validation. It should, therefore, be noted that some items may be interpreted differently. For instance, some coaches indicating ‘use’ of the 11+ could only have performed parts of the exercises, adopted a different progression, or selected another number of sets/repetitions. In sum, building on our data, future research should aim to more precisely define and delineate the different factors contributing to constructs such as program usage, feasibility or suitability.

Data were collected from German football coaches only. Although the results could apply to other European countries, they may not be valid for others. The limited evidence available indicates considerable regional differences between countries such as Germany or Australia and Saudi Arabia or Nigeria. Additionally, due to the sports-related organizational differences [29], e.g., regarding league system and license education, it seems paramount to conduct further research with a multinational focus.

With 16%, our response rate was relatively low. However, for three reasons, a non-responder bias substantially impacting the results can almost be excluded. While the number of undelivered e-mails was documented, it is unclear whether all coaches read the email containing the invitation to participate. Thus, the true response rate might have been higher. Also, according to the wave analysis, there was no significant difference between early and late responders. Finally, a higher response rate and measures to increase the response rate have been demonstrated not to produce more accurate results [30,31].

## Conclusions

Slightly less than half of the surveyed football coaches were aware of the 11+. Most of the coaches who knew the intervention reported applying it regularly. Not using the programme rather seemed to be related to a lack of awareness than to a low perceived feasibility or suitability. While this hypothesis still needs to verified by future research, the development of more effective communication and implementation strategies, for example, during license education, is crucial in order to increase the usage of the programme.

## References

[1] Leppänen M, Aaltonen S, Parkkari J, Heinonen A, Kujala UM. Interventions to Prevent Sports Related Injuries: A Systematic Review and Meta-Analysis of Randomised Controlled Trials. *Sports Med*. 2014; 44(4):473–86. doi: 10.1007/s40279-013-0136-8 24370993 10.1007/s40279-013-0136-8

[2] Fradkin AJ, Zazryn TR, Smoliga JM. Effects of Warming-up on Physical Performance: A Systematic Review With Meta-analysis. *J Strength Cond Res*. 2010; 24(1):140–8. doi: 10.1519/JSC.0b013e3181c643a0 19996770 10.1519/JSC.0b013e3181c643a0

[3] Póvoas SC, Seabra AF, Ascensão AA, Magalhães J, Soares JM, Rebelo AN. Physical and Physiological Demands of Elite Team Handball. *J Strength Cond Res*. 2012; 26(12):3365–75. doi: 10.1519/JSC.0b013e318248aeee 22222325 10.1519/JSC.0b013e318248aeee

[4] Castellano J, Blanco-Villaseñor A, Álvarez D. Contextual Variables and Time-Motion Analysis in Football. *Int J Sports Med*. 2011; 32(06):415–21. 21590641 10.1055/s-0031-1271771

[5] Matthew D, Delextrat A. Heart rate, blood lactate concentration, and time–motion analysis of female basketball players during competition. *J Sport Sci*. 2009; 27(8):813–21. 10.1080/02640410902926420 19551549

[6] Steffen K, Meeuwisse WH, Romiti M, Kang J, McKay C, Bizzini M et al Evaluation of how different implementation strategies of an injury prevention programme (FIFA 11+) impact team adherence and injury risk in Canadian female youth football players: a cluster-randomised trial. *Br J Sports Med*. 2013; 47(8):480–7. doi: 10.1136/bjsports-2012-091887 23486935 10.1136/bjsports-2012-091887

[7] Soligard T, Myklebust G, Steffen K, Holme I, Silvers H, Bizzini M et al Comprehensive warm-up programme to prevent injuries in young female footballers: cluster randomised controlled trial. *BMJ* 2008; 337. 10.1136/bmj.a2469 PMC2600961 19066253

[8] Barengo N, Meneses-Echávez J, Ramírez-Vélez R, Cohen D, Tovar G, Bautista J. The Impact of the FIFA 11+ Training Program on Injury Prevention in Football Players: A Systematic Review. *Int J Environ Pub Health* 2014; 11(11):11986–2000. 10.3390/ijerph111111986 PMC4245655 25415209

[9] Al Attar WS, Soomro N, Pappas E, Sinclair PJ, Sanders RH. How effective are F-MARC injury prevention programs for soccer players? A systematic review and meta-analysis. *Sports Med*. 2016; 46(2):205–217. doi: 10.1007/s40279-015-0404-x 26403470 10.1007/s40279-015-0404-x

[10] Thorborg K, Krommes KK, Esteve E, Clausen MB, Bartels EM, Rathleff MS. Effect ofspecific exercise-based football injury prevention programmes on the overall injury rate in football: a systematic review and meta-analysis of the FIFA11 and 11+ programmes. *Br J Sports Med*. 2017; 51(7):562–571. doi: 10.1136/bjsports-2016-097066 28087568 10.1136/bjsports-2016-097066

[11] Steffen K, Emery CA, Romiti M, Kang J, Bizzini M, Dvorak J et al High adherence to a neuromuscular injury prevention programme (FIFA 11+) improves functional balance and reduces injury risk in Canadian youth female football players: a cluster randomised trial. *Br J Sports Med*. 2013; 47(12):794–802. doi: 10.1136/bjsports-2012-091886 23559666 10.1136/bjsports-2012-091886

[12] Soligard T, Nilstad A, Steffen K, Myklebust G, Holme I, Dvorak J et al Compliance with a comprehensive warm-up programme to prevent injuries in youth football. *Br J Sports Med*. 2010; 44(11):787–93. doi: 10.1136/bjsm.2009.070672 20551159 10.1136/bjsm.2009.070672

[13] Bizzini M, Junge A, Dvorak J. Implementation of the FIFA 11+ football warm up program: How to approach and convince the Football associations to invest in prevention. *Br J Sports Med*. 2013; 47(12):803–6. doi: 10.1136/bjsports-2012-092124 23813485 10.1136/bjsports-2012-092124 PMC3717809

[14] Bizzini M, Junge A, Dvorak J. FIFA 11+ Injury Prevention in Amateur Football from Development to Worldwide Dissemination In: Kanosue K, Ogawa T, Fukano M, Fukubayashi T, editors. Sports Injuries and Prevention. Tokyo: Springer Japan; 2015 p. 199–208.

[15] Bizzini M & Dvorak J. FIFA 11+: an effective programme to prevent football injuries in various player groups worldwide—a narrative review. Brit J Sports Med. 2015;49(9):577–579. 25878073 10.1136/bjsports-2015-094765 PMC4413741

[16] Owoeye OBA, Akinbo SRA, Olawale OA, Tella BA, Ibeabuchi NM. Injury prevention in football: Knowledge and behavior of players and availability of medical care in Nigerian youth football league. *S Afr J SM*. 2013; 25(3):77–80.

[17] Al Attar WS, Soomro N, Sinclair PJ, Pappas E, Muaidi QI, Sanders RH. Implementation of an evidence-based injury prevention program in professional and semi-professional soccer. *Sports Sci Coach*. 2017, 0(0):1–9.

[18] O’Brien J & Finch CF. Injury prevention exercise programmes in professional youth soccer: understanding the perceptions of programme deliverers. *BMJ Open Sport Exerc Med*. 2016; 2:e000075 doi: 10.1136/bmjsem-2015-000075 27900158 10.1136/bmjsem-2015-000075 PMC5117035

[19] Kelley K, Clark B, Brown V, Sitzia J. Good practice in the conduct and reporting of survey research. *Int J Qual Health Care* 2003; 15(3):261–6. 12803354 10.1093/intqhc/mzg031

[20] Miller L.E. SK. Handling Nonresponse Issues. J Ext. 1983; (9/10):45–50.

[21] Lewis EF, Hardy M, Snaith B. Estimating the Effect of Nonresponse Bias in a Survey of Hospital Organizations. *Eval Health Prof*. 2013; 36(3):330–51. doi: 10.1177/0163278713496565 23908382 10.1177/0163278713496565

[22] Junge A, Lamprecht M, Stamm H, Hasler H, Bizzini M, Tschopp M, Reuter H, Wyss H, Chilvers C, Dvorak J. Countrywide campaign to prevent soccer injuries in Swiss amateur players. Am J Sports Med. 2011;39(1):57–63. doi: 10.1177/0363546510377424 20956263 10.1177/0363546510377424

[23] Deutscher Fussball-Bund. Trainerausbildung. 2015 June 14 [cited 11 January 2018]. Available from: https://www.dfb.de/sportl-strukturen/trainerausbildung/.

[24] Norcross MF, Johnson ST, Bovbjerg VE, Koester MC, Hoffman MA. Factors influencing high school coaches’ adoption of injury prevention programs. J Sci Med Sport. 2016;19(4):299–304. doi: 10.1016/j.jsams.2015.03.009 25866072 10.1016/j.jsams.2015.03.009

[25] McKay CD, Steffen K, Romiti M, Finch CF, Emery CA. The effect of coach and player injury knowledge, attitudes and beliefs on adherence to the FIFA 11+ programme in female youth football. *Br J Sports Med*. 2014; 48(17):1281–6. doi: 10.1136/bjsports-2014-093543 24928848 10.1136/bjsports-2014-093543

[26] Joy EA, Taylor JR, Novak MA, Chen M, Fink BP, Porucznik CA. Factors influencing the implementation of anterior cruciate ligament injury prevention strategies by girls soccer coaches. J Strength Condit Res. 2013;27(8):2263–2269. 10.1519/JSC.0b013e31827ef12e 23287828

[27] McKay C, Merrett C, Emery C. Predictors of FIFA 11+ Implementation Intention in Female Adolescent Football: An Application of the Health Action Process Approach (HAPA) Model. *Int J Environ Pub Health* 2016; 13(7):657. 10.3390/ijerph13070657 PMC4962198 27399746

[28] Saunders N, Otago L, Romiti M, Donaldson A, White P, Finch C. Coaches' perspectives on implementing an evidence-informed injury prevention programme in junior community netball. *Br J Sports Med*. 2010; 44(15):1128–32. doi: 10.1136/bjsm.2009.069039 20542975 10.1136/bjsm.2009.069039

[29] Fort R. European and North American Sports Differences(?). *Scot J Polit Econ*. 2000; 47(4):431–55.

[30] Curtin R, Presser S, Singer E. The effects of response rate changes on the index of consumer sentiment. *Public Opin Q*. 2000; 64(4):413–28. 11171024 10.1086/318638

[31] Keeter S, Kennedy C, Dimock M, Best J, Craighill P. Gauging the Impact of Growing Nonresponse on Estimates from a National RDD Telephone Survey. *Public Op Quarterl*. 2006; 70(5):759–79.

